# From structure to experience: a Spinozist framework for consciousness rooted in critical brain dynamics

**DOI:** 10.1093/nc/niag066

**Published:** 2026-10-26

**Authors:** Inbal Algom, Yehonatan Nachshoni, Moti Salti, Oren Shriki

**Affiliations:** Department of Industrial Engineering and Management, Ben-Gurion University of the Negev, 1 David Ben-Gurion Blvd., P.O. Box 653, Beer-Sheva 8410501, Israel; School of Brain Sciences and Cognition, Ben-Gurion University of the Negev, 1 David Ben-Gurion Blvd., P.O. Box 653, Beer-Sheva 8410501, Israel; Department of Jewish History, Ben-Gurion University of the Negev, 1 David Ben-Gurion Blvd., P.O. Box 653, Beer-Sheva 8410501, Israel; Department of Industrial Engineering and Management, Ben-Gurion University of the Negev, 1 David Ben-Gurion Blvd., P.O. Box 653, Beer-Sheva 8410501, Israel; School of Brain Sciences and Cognition, Ben-Gurion University of the Negev, 1 David Ben-Gurion Blvd., P.O. Box 653, Beer-Sheva 8410501, Israel; Department of Industrial Engineering and Management, Ben-Gurion University of the Negev, 1 David Ben-Gurion Blvd., P.O. Box 653, Beer-Sheva 8410501, Israel; School of Brain Sciences and Cognition, Ben-Gurion University of the Negev, 1 David Ben-Gurion Blvd., P.O. Box 653, Beer-Sheva 8410501, Israel

**Keywords:** consciousness, theories and models, philosophy, computational modeling, perception

## Abstract

The study of consciousness is divided between mechanistic accounts that overlook subjective experience and phenomenological approaches limited to neural correlates. We propose a unified framework by grounding our ConCrit model of consciousness and critical brain dynamics within Spinoza’s ontological monism. Spinoza’s principle of psycho-physical parallelism—mind and body as attributes of one substance—provides a metaphysical basis to dissolve the explanatory gap between neural processes and lived experience. Within this framework, three properties which are maximized near criticality correspond, under Spinoza’s parallelism, to mental dimensions: entropy corresponds to experiential richness—the diversity of idea under the attribute of Thought, internal susceptibility to reflexive capacity or degrees of autonomy, and scale-free dynamics to the structural alignment with the common order of nature that, under the attribute of Thought, is expressed in adequate ideas. Thus, we propose that brain activity near criticality under the attribute of Extension is the very same phenomenon that, under the attribute of Thought, is conscious experience. This synthesis reinterprets leading theories—including Integrated Information Theory, Global Neuronal Workspace Theory, and the Entropic Brain Hypothesis—as complementary views of a shared underlying reality. By situating ConCrit within Spinoza’s monism, we argue that consciousness is not emergent or epiphenomenal but the parallel manifestation of universal organizational principles, offering a conceptually unified and interpretively transparent framework for neuroscience.

## Introduction

The contemporary scientific study of consciousness is torn between two conflicting approaches. One focuses on a mechanistic description of consciousness, often accompanied by an ontological dismissal of qualia—subjective experience—framing it as an epiphenomenon or mere byproduct of neurophysiological activity (Chalmers 2017). In contrast, other researchers investigate subjective experience and its neurophysiological basis, yet remain limited to describing the neural correlates of consciousness (NCC) mostly neglecting subjective experience itself (Seth and Bayne 2022).

Both approaches are deeply rooted in Western philosophy. Mechanistic descriptions of consciousness can be traced back to ancient Greek philosophy (e.g. Plato’s Phaedo) and gained new ground at the dawn of the scientific revolution. Galileo Galilei argued that science should describe the world through quantifiable, objective properties and explicitly excluded subjective experience from its domain (Goff 2019). The “hard problem of consciousness,” a term coined by David Chalmers along with the complementary “easy problem,” asks how subjective experience arises from neurophysical processes (Chalmers 1995, 2017). It emerged in the early modern period as a development of what Chalmers calls the “easy problem,” the psychophysical problem formulated by Descartes in the 17th century. Descartes introduced a foundational ontological split between mind and body, and more broadly between consciousness and the physical world. Yet the deeper roots of this divide reach back to the ancient Greek binary distinction between mind and matter—a conceptual boundary that still shapes contemporary discourse (Ostenfeld 2018).

The decisive shift occurred within the context of the scientific revolution, which entrenched the split between empirical science and philosophical inquiry. Since then, science and philosophy have evolved largely along parallel, non-intersecting tracks: as science advanced our knowledge of the physical world and the body, consciousness remained conceptually elusive. The broader scientific tradition continued along the Cartesian path, with the metaphysical division persisting implicitly in neuroscience (Heil 1992, Hendriksen 2009, Sanguineti 2015, Berrios 2018, Bennett and Hacker 2022, Barbara 2024). This trajectory culminated in 20th-century efforts to isolate the neural basis of consciousness. In 1990, Crick and Koch proposed that identifying NCCs could provide a concrete framework for studying consciousness scientifically (Crick and Koch 1990). Yet even in their seminal paper, they advised leaving the question of qualia aside “at the moment.” Consistent with this view, leading theories such as Integrated Information Theory (IIT) and Global Neuronal Workspace Theory (GNWT) focus on describing the mechanisms underlying consciousness while paying less attention to its subjective qualities. IIT describes physical structures known as “complexes,” while GNWT describes physical processes such as “ignition” that correlate with conscious states (Tononi 2004, Mashour et al. 2020). While IIT begins with axioms rooted in phenomenology and GNWT centers on mechanisms of access consciousness, both theories face challenges in explaining why or how the physical structures they describe yield subjective experience, and in clearly defining what exactly “consciousness” means.

Neuroscience has made significant progress in uncovering the mechanisms underlying cognitive and behavioral functioning, but it has made little headway on qualia and the hard problem. Contemporary theories of consciousness address different aspects of this problem. For example, in GNWT, information becomes consciously accessible when it is globally broadcast across the brain, sustained by recurrent ignition of neural activity across connected regions (Mashour et al. 2020). IIT, in contrast, more directly addresses the structure of subjective experience conceptualizing qualia as emerging from the informational structure of a multidimensional “qualia space”—each experience corresponding to a specific shape within this space (Tononi 2004). Yet neither GNWT nor IIT resolves the transition from physical mechanisms to subjective experience, nor do they present empirical evidence supporting the assumption that consciousness arises solely from material processes. While these frameworks describe neural correlates, they do not explain why such correlates produce the qualitative dimension of experience—the explanatory gap remains.

The notion of this explanatory gap emerged as a central challenge in philosophy of mind through the works of Thomas Nagel and Joseph Levine. Nagel’s influential essay “What Is It Like to Be a Bat?” (1974) underscored the fundamental divide between first-person subjective experience and third-person scientific description, arguing that no physical account can fully capture what it is like to have a particular conscious experience (Nagel 1974). Building on this, Levine (1983) coined the term “explanatory gap” to highlight the persistent difficulty physicalist theories face in explaining how objective neural processes give rise to qualitative states (Levine 1983). For Levine, the gap reflects an inherent limit to physical explanation: knowing all physical facts still does not yield an account of why or how specific brain states correlate with subjective experience.

Some theorists, such as Dennett, reject this gap by arguing for an equivalence between qualia and their underlying mechanisms. Dennett suggests that our confusion about qualia stems from our incomplete understanding of these mechanisms: once the physical mechanisms are revealed, qualia are “demystified.” (Dennett 2016, 2018). He compares this to stage magic—the brain creates the illusion of phenomenality, and understanding the “trick” removes its mystery. Yet this remains controversial and is often criticized for downplaying the lived reality of subjective experience.

Neurophenomenology, developed by Francisco Varela, provides a clear model for combining subjective experience with empirical brain research, supported by stable philosophical foundations (Varela 1996, Berkovich-Ohana et al. 2020). It links first-person reports with third-person data through structured phenomenological methods and demonstrates that neuroscience benefits from a strong philosophical basis. Its core approach brings together empirical neuroscience, phenomenology as a theory of knowledge and as a methodology for analyzing subjective experience, as well as insights from Buddhist contemplative traditions. By doing so, neurophenomenology has paved the way for integrating first-person experience and empirical evidence within a coherent philosophical framework. In this way, it shows how careful philosophical grounding can expand the study of consciousness beyond standard empirical limits. Yet, despite this advance, it remains within a psychophysical dualism and tied to the search for NCC (Petitot 1999, Bayne 2004, Overgaard 2004, Bockelman et al. 2013). For this reason, we see it as a valuable starting point that highlights the need for a deeper, unified philosophical framework.

This persistent gap, and the lack of a shared framework among researchers has left the field at an impasse and created a paradigmatic deadlock. Advancing beyond this requires a careful re-examination of the paradigm itself, its underlying logical structure, and the historical trajectory through which these assumptions have evolved. By clarifying these foundations, we can assess whether the scientific method and its inherited conceptual framework can adequately address this deadlock or whether an alternative perspective is required.

Within the Western tradition, two major figures stand out as conceptual rebels: Aristotle and Spinoza. Aristotle’s principle of hylomorphism holds that matter and form are inseparable aspects of any existing thing. Spinoza extended this line of thought, offering a unified metaphysical account in which mind and body, consciousness and the physical, ontology, and epistemology are parallel attributes of a single substance. He formulates this view throughout the “Ethics,” describing everything as existing simultaneously under the attributes of thought and extension. In doing so, Spinoza upheld the Aristotelian tradition of hylomorphism while grounding it in his own radical vision of ontological unity.

Spinoza is indispensable to any philosophical and scientific discussion of consciousness in general, and of qualia in particular. He stands as the first major post-Cartesian natural philosopher, rooted in the dawn of modern Western thought yet drawing on non-dualistic Jewish and Neoplatonic medieval traditions. Some scholars even regard his entire philosophical project as aimed at establishing the constitution of the free subject (Brykman 1972, Yovel 1989, Yovel and Segal 2004). In his Ethics, Spinoza argues that all of nature expresses a single substance through two fundamental attributes: extension (the physical) and thought (the mental). These are not separate substances but two aspects of one reality. Thus, mind and body are not distinct entities that causally interact but rather parallel expressions of the same underlying structure. He illustrates this with the example of a circle: the physical form (extension) and the idea of the circle (thought) are two perspectives on the same object. As he writes, “the order and connection of ideas is the same as the order and connection of things.” This principle, known as psycho-physical parallelism, implies that for every physical structure, there is a corresponding mental representation—but not as two separate realms, rather as two inseparable aspects of the same substance. This unified view offers a compelling alternative to both dualistic and reductionist models. Instead of positing a causal link from brain activity to subjective experience, Spinoza’s framework suggests an ontological identity: the mental and physical are two sides of the same coin. Conscious experience, then, is not a mysterious byproduct but the mental expression of the same underlying substance.

Recent work has proposed integrative frameworks that attempt to bridge neural dynamics and subjective experience by identifying shared structural principles across domains. Notably, the Temporo-Spatial Theory of Consciousness (TTC) emphasizes the brain’s intrinsic temporal and spatial organization as a fundamental substrate linking neuronal activity and phenomenological features. In this view, temporo-spatial dynamics function as a “common currency” through which neural and mental features are related (Northoff and Zilio 2022, Northoff et al. 2025). This approach shares with the present work the goal of moving beyond isolated neural correlates toward a more integrative and non-reductionist account of consciousness. This position differs in an important respect from the “common currency” hypothesis proposed within the TTC. While TTC formulates the relationship between neural and experiential domains in terms of shared temporo-spatial structure, it does so primarily at the level of empirically grounded correspondence (Northoff and Zilio 2022, Northoff et al. 2025). In contrast, the present framework adopts a monistic identity claim: neural dynamics and conscious experience are not merely structurally aligned but are the same underlying reality expressed under different attributes. In this sense, the framework provides an ontological grounding for why such shared structural principles arise.

Since current frameworks may be insufficient to fully explain consciousness, there is an urgent need for novel approaches that reconcile its subjective nature with objective scientific inquiry. Echoing Nagel’s critique, we propose a perspective that investigates consciousness directly rather than focusing exclusively on brain mechanisms. In this context, Spinoza’s insight—that mind and matter are distinct yet parallel attributes of a single underlying reality—offers crucial advantages. It bridges the explanatory gap by integrating subjective experience with physical processes, supports a holistic perspective in which mental and physical domains represent complementary viewpoints of the same phenomenon, and anchors consciousness research within a robust philosophical foundation.

In subsequent sections, we introduce the concept of critical brain dynamics, presenting it as a neurodynamical framework describing the physical organization of brain activity associated with conscious experience—termed the ConCrit framework. While approaches such as TTC identify shared structural principles linking neural and experiential domains, they primarily describe correspondences across levels (Northoff and Zilio 2022). In contrast, the present framework combines a dynamical account of brain activity near criticality with a Spinozist ontological perspective, thereby providing both a candidate mechanism and a metaphysical grounding for why such correspondences arise.

## Redefining consciousness through Spinoza’s prism

The fundamental principle of Spinoza’s philosophy is the idea that all that exists forms one unified whole—a monistic reality that appears in different modes or forms. As he states: “only one substance can be granted in the universe” (Spinoza, Ethics, Book 1, Proposition 14, Corollary 1 [hereafter: E1P14C1]).

To understand the logic of this claim, it is essential to clarify two core concepts that run throughout the “Ethics”: substance and attribute. Drawing on a tradition that reaches back to Greek thought, and developed by Jewish and Islamic philosophers, (Hartman 1976, Harvey 1981, Donagan 1991, Tabatabaei 2010, Melamed 2013) a substance is the defining essence of a thing, the characteristic without which it would not be what it is (*cf.*  Aristotle 1984a; 1984b). For Spinoza, however, an attribute is the way the intellect perceives and describes this substance. Substance is that which exists in and through itself (E1Def3), while attributes are its real expressions—what the intellect perceives as constituting its essence (E1Def4; cf. Shein 2009)—not merely subjective forms of apprehension, but the very ways in which the one substance is and is intelligible.

As Spinoza puts it:

By substance I mean that which is in itself, and is conceived through itself: in other words, that of which a conception can be formed independently of any other conception. (E1Def3)

By attribute I mean that which the intellect perceives as constituting the essence of substance. (E1Def4)

Substance and attributes are thus not separate entities but two aspects of one and the same reality. Indeed, Spinoza insists that there is not a plurality of substances in the world; all things are simply expressions—attributes or modes—of the one substance. The chair on which I sit, the tree outside my window, the human brain, all are the one substance appearing as chair, tree, or brain. Put differently, there is a chair-substance, a tree-substance, and a brain-substance, but ultimately, they are one.

Spinoza maintains that this single substance appears to us primarily under two fundamental attributes: Thought and Extension. Thought is the essence of anything that can be known as mind; Extension is its presence in space. For Spinoza, the mode of being in thought is no less real than the mode of being in extension (Nadler 2006, Galili 2024: 201). As he writes: “Mind and body are one and the same thing, conceived now under the attribute of thought, now under the attribute of extension.” (E2P7S). Likewise: “The mind and the body are one and the same thing, which is conceived now under the attribute of thought, now under the attribute of extension.” (E3P2S; Spinoza 1995, cf. Letters, Letter 9; Galili 2024, Chapter 5).

Spinoza’s fundamental distinction, in our context, between body and mind is that a body is something extended in space, whereas the mind is something that thinks. By “thinking,” he refers to mental activity of any kind. In Spinoza’s parallelism, bodily state has a corresponding mental idea (E2P13; Nadler 2008). Degrees of reflexive integration vary with bodily complexity. Following Spinoza, and in order to align with the prevailing discourse in consciousness studies, we define: Consciousness refers to any mental aspect of any bodily activity. This does not entail that every such mental state is reflexively available to the mind as a whole. The degree of reflexive awareness varies with the structural organization of the mind–body system, as elaborated in the sections that follow.

The question of whether Spinoza has a theory of consciousness, and if so what it consists in, has been the subject of sustained scholarly debate over the past two decades. Three issues are especially relevant here: first, whether consciousness is a graded feature of mental life or a non-graded technical notion; second, whether its primary locus is the overall organization of the mind–body system or the content of particular ideas; and third, whether Spinoza’s use of conscientia maps onto what contemporary philosophy of mind calls “consciousness.” A substantial body of interpretation reads Spinoza as committed to a graded conception of mental activity tied to bodily complexity, adequacy, causal autonomy, or the power of acting, developed in different directions by Curley, Nadler, Sangiacomo, Della Rocca, Garrett, Marshall, and Shein (2015). At the same time, other interpreters either adopt a more access-oriented reading of conscientia (Marrama 2017, Toth 2026) or question whether Spinoza has a unified theory of consciousness in the contemporary sense at all (LeBuffe 2010, Garber 2021). We do not claim to settle these interpretive disputes here. Rather, we adopt a selective reading that is sufficient for the present project: one on which mental activity exists along a continuum, and on which the structure of that activity is inseparable from the organization of the body that underlies it.

This framework allows us to distinguish, without separating, two levels of explanation. On the one hand, the global dynamical organization of the brain–body system under the attribute of Extension determines the system’s capacity for integration, differentiation, and reflexive modulation. On the other hand, the specific content of a given mental activity shapes how that capacity is actualized in a particular case. Spinoza’s remark that an idea of a bodily affection tells us more about our own body than about the external object (E2P16C2) remains central here: the quality of experience is not fixed by the external cause alone, but by the way the affection is internally organized and represented within the system. The contribution of the present framework lies in proposing a contemporary operationalization of the bodily side of this relation, addressing the question of how the relevant kind of bodily complexity should be measured—a question Nadler (2008) identified as outstanding—by treating proximity to criticality, scale-free organization, and entropy-related variability as candidate measures of the structural complexity Spinoza associates with greater power of the mind.

Spinoza’s classic statement, “the order and connection of ideas is the same as the order and connection of things” (E2P7S), expresses a strict correspondence between mental and physical organization. This correspondence may be understood as a form of structural isomorphism between neural and mental features. However, the term is not used here in a reductive sense, as implying a one-to-one mapping between isolated neural states and discrete subjective experiences, nor as a causal relation between two separate ontological domains. Rather, following Spinoza’s psycho-physical parallelism, “isomorphism” refers to a correspondence in relational organization between neural and mental features, understood as two expressions of the same underlying reality under the attributes of Thought and Extension. This is psycho-physical parallelism—not the coexistence of two separate realms but a single reality understood under two attributes.

Thus, the mind–body “interaction” is a non-problem: mind and body are the same thing described under different aspects (Bennett 1984, Mason 1999). From a human perspective, the body is the object of the mind, and the mind is the idea of the body (E2P21 and Scholium). They exist only in relation to each other, just as all things do—simultaneously as idea and extension. The classic mind–body problem, in which mental and physical states are seen to interact causally, simply does not arise for Spinoza. For instance, where a dualist sees the state “I am hungry” as a physiological condition plus a separate feeling, Spinoza sees only one unified state: “hunger” is at once physiological (extension) and mental (thought). The same holds for perception: when I sit on a chair, there is the physical contact of body and chair (extension) and the idea of the chair (thought). Unlike Descartes, who posits two distinct substances—*res cogitans* and *res extensa*—Spinoza argues there is only one. In Descartes’ framework, the mental and the physical require an external mediator (like the pineal gland) to bridge them (Descartes 2016). For Spinoza, no such *deus ex machina* is needed; the mental and the physical are not distinct substances but expressions of the same underlying substance, conceived under different attributes which are distinguished only by the human intellect (Galili 2024: 205).

This unified view dissolves the fundamental epistemological split that still structures much of today’s philosophy and neuroscience: the divide between ontology and epistemology, between object and phenomenology. In Spinoza’s prism, all that exists is simultaneously object and phenomenon, being and knowing. The human being is not exceptional within this unity, except in the degree of its cognitive complexity, which appears in the intricacy of its ideas (thought) and the structure of its body (extension). From a contemporary scientific point of view, Spinoza’s thought does not stop an abstract philosophical theorizing consciousness. By rooting the relation between structure and experience in this monistic framework, Spinoza opens a path for rethinking consciousness: the more complex a system is in the attribute of extension, the more complex—and potentially conscious—is its corresponding idea in the attribute of thought. In this way, Spinoza lays a foundation for a scientifically informed yet non-reductive account of consciousness.

We build on this perspective by linking Spinoza’s key propositions to three physical properties of brain activity—entropy, internal susceptibility, and scale-free dynamics (summarized in Table 1)—which correspond to phenomenal experience, reflexivity, and structural alignment that underlies the formation of adequate ideas. These properties become most pronounced when the brain operates near criticality, a regime at the edge of a phase transition, where small parameter changes can trigger large-scale transformations in brain dynamics and activity patterns.

**Table 1:** Physical-mental correspondences in a Spinozist framework

**Physical property (extension)**	**Mental expression (thought)**
Entropy	Experiential richness—the diversity and breadth of possible subjective states
Internal susceptibility	Reflexive capacity—the mind’s capacity for self-monitoring and autonomous self-regulation
Scale-free dynamics (Fractal structure)	Structural adequacy—alignment with the common order of nature, enabling generalized understanding

### Complexity, entropy, and the richness of experience

A widely discussed distinction in consciousness studies is that between phenomenal consciousness and access consciousness (Block 1995). According to Block’s distinction, phenomenal consciousness refers to the subjective quality of experience, “what it is like to be” in a given mental state, often labeled *qualia*. Access consciousness refers to mental contents available for reasoning, verbal report, and the guidance of action. Block proposed that phenomenal consciousness exceeds access consciousness in richness, encompassing experiences that are not cognitively accessible or linguistically expressible, and the empirical dissociability of the two remains contested (Schlicht 2012).

While methodologically useful, this distinction reflects a dualistic residue within contemporary theory: it presupposes that the qualitative character of experience can be functionally separated from its availability to reasoning and report. From a Spinozist standpoint, this presupposition is neither necessary nor coherent. Rather than positing two kind of consciousness that must subsequently be reconciled, we propose three structural dimensions of a single conscious activity—experiential richness, reflexivity, and structural alignment with the common order of nature—each of which becomes most pronounced as the brain approaches criticality.

From a Spinozist perspective, Block’s dichotomy fail to rise in the first place, or at least dissolves into a more integrated framework. Because mind and body are parallel expressions of one substance, every mental activity is intrinsically both qualitative and available to the mind—though to varying degrees. What differs across mental states is not the presence or absence of “phenomenal” or “access” dimension, but the degree to which the underlying activity is rich, reflexive, and structurally coherent. According to our reading, phenomenal and access consciousness are not distinct systems but rather two inseparable aspects of a single psychophysical event. As shown earlier, Spinoza systematically affirms a strict ontological monism, where mental and physical attributes are parallel expressions of one and the same substance. Accordingly, consciousness always has both a phenomenal and an access dimension, though the degree and adequacy of access may vary. Spinoza writes:

Whatever happens in the object of the idea constituting the human Mind must be perceived by the human Mind, or there will necessarily be an idea of that thing in the Mind. (E2P12)

Yet,

The idea of the idea of any affection of the human Body does not involve adequate knowledge of the human Mind. (E2P29)

In Spinozistic terms, any phenomenal state necessarily involves a corresponding idea in the mind, even if it lacks *adequate* (i.e. fully accessible or self-reflective) representation. A phenomenal state without full access may thus be understood as an “inadequate idea,” shaped by passive associations rather than by understanding things through their proper causal order (E2P7). Here, “understanding things” refers to the formation of adequate ideas: ideas that grasp their objects through their causal relations, rather than through partial, passive, or merely associative impressions. The richness or poverty of such access depends, in part, on the overall complexity and organization of the mind–body system at a given moment.

Spinoza’s view allows us to reconceive consciousness as comprising multiple structural dimensions, each with both mental and physical expressions. The dimension of experiential richness arises in proportion to the body’s power to act and be acted upon. As Spinoza puts it:

In proportion as a body is more capable than others of doing many things at once, or being acted on in many ways at once, so its mind is more capable than others of perceiving many things at once. (E2P13S)

This proportional language should be understood as indicating a graded positive relation between bodily organization and experiential richness, not a simple linear mapping between neural complexity and realized experience.

And,

The human mind is capable of perceiving a great many things, and is the more capable, the more its body can be disposed in a great many ways. (E2P14)

Thus, as the physical complexity of the body increases, so too does the mental complexity of its corresponding idea. Consciousness, in this view, is not a binary property that one either has or lacks, but it has degrees or levels in proportion to the structure of the mind–body system. Consciousness unfolds along a continuum, with its degree reflecting the complexity of the mind–body system. Steven Nadler (2008) articulates this point succinctly: “Human or higher consciousness for Spinoza is nothing but the mental correlate of the superlative complexity of the human body.” A stone, while possessing an idea, lacks reflective awareness, since its bodily simplicity corresponds to an impoverished field of mental life; the human body’s dynamic richness corresponds to a rich field of consciousness (Nadler 2008).

Interestingly, this view finds resonance in contemporary neuroscience. Here, the complexity of brain activity, often measured in terms of entropy, correlates with the richness of conscious experience. More specifically, the relevant notion here is Shannon entropy—quantifying the diversity of distinguishable neural activity patterns a system can express, i.e. its representational capacity. A brain with more dynamic and flexible connectivity patterns exhibits higher entropy, reflecting a broader repertoire of experiential states (Carhart-Harris et al. 2014, Carhart-Harris 2018, Toker et al. 2022).

Entropy is also tightly linked to susceptibility—a system’s sensitivity to internal or external perturbation. Near criticality, systems display maximal susceptibility: even minor inputs can yield large-scale shifts in behavior. In formal models, maximizing entropy coincides with maximizing information responsiveness (Shriki et al. 2000, Shriki and Yellin 2016). A brain operating in such a regime produces a wider range of outputs, corresponding under the attribute of Thought to richer experiential content. This claim is supported by empirical studies: unconscious states such as deep sleep, anesthesia, and coma are marked by decreased entropy and diminished experiential variety (Comolatti et al. 2019). Conversely, some altered states of consciousness, such as those induced by meditation and psychedelics, are consistently associated with increased entropy and expanded experiential content (Carhart-Harris et al. 2014, Carhart-Harris 2018, Toker et al. 2022, Atad et al. 2025).

Building on these findings, Carhart-Harris advanced the Entropic Brain Hypothesis (EBH), which proposes that the quality of conscious states is indexed by brain entropy. Normal waking consciousness occupies an intermediate zone—structured enough for stability yet flexible enough for vivid experience. Psychedelics elevate entropy within this range, while sedation and anesthesia lower it (Carhart-Harris et al. 2014, Carhart-Harris 2018).

Complementing this view, IIT offers a structural account of consciousness grounded in the causal relationships within a system. Among its central axioms is cause-effect power, the system’s overall capacity to influence and be influenced by itself (Tononi et al. 2016). In our framework, experiential richness, indexed by entropy, corresponds to this cause-effect power.

This interpretation aligns naturally with Spinoza’s propositions: the more structured and responsive a body is, the more complex and richer is its corresponding idea. In this sense, entropy provides a contemporary scientific vocabulary for articulating what Spinoza inferred through rational metaphysics: the gradations of conscious experience mirror the gradations of physical organization.

It is important to clarify that entropy is not proposed here as a standalone mechanism of consciousness, nor as a generic measure of randomness. Rather, in the present context, we specifically refer to Shannon entropy as a measure of representational capacity—the diversity of structured neural activity patterns that a system can express in response to inputs. This notion captures the capacity of the system to explore a rich and differentiated repertoire of internal states, which we propose to be a key constraint on the level and richness of conscious experience. Importantly, this capacity reflects a *potential*: even if only a subset of states is realized at a given moment, the size and organization of the accessible state space determine the range of possible experiences the system can support. In this sense, representational entropy serves as a proxy for the richness of conscious experience, not through a one-to-one mapping between neural states and specific experiences, but through the breadth and flexibility of the representational landscape.

This notion provides the clearest quantitative bridge to Spinoza’s claim that the mind is more capable insofar as the body can “do many things at once” or “be disposed in a great many ways”: the greater the number of structured configurations available to the system, the greater its capacity for differentiated experience.

Lempel-Ziv complexity estimates the complexity of a finite sequence by counting the number of distinct patterns required to describe it—capturing how incompressible, and therefore how diverse, a specific signal is (Lempel and Ziv 1976, Casali et al. 2013). Lempel Ziv Complexity (LZC) has been prominently applied in consciousness research, most notably in the Perturbational Complexity Index(Casali et al. 2013), which uses normalized LZC of TMS-evoked spatiotemporal patterns to discriminate levels of consciousness. However, LZC quantifies pattern diversity within a specific signal or response, whereas Shannon entropy of the neural repertoire captures the system’s capacity—the full space of possible states it can generate across inputs. These are related but conceptually distinct: a system may produce a highly complex individual response without necessarily possessing a large overall repertoire of accessible states.

Within the ConCrit framework, representational entropy is tightly linked to susceptibility and is expected to be enhanced in the vicinity of critical dynamics, where the system optimally balances stability and flexibility. Near criticality, neural systems maximize their responsiveness to both external inputs and internal fluctuations, thereby expanding the repertoire of accessible states. Deviations from this regime, whether toward overly rigid (subcritical) or overly dominated (supercritical) dynamics, are expected to reduce this effective repertoire, albeit through different mechanisms. Thus, entropy, in this specific and constrained sense, expresses the parallel between the dynamical organization of neural systems and the graded richness of conscious experience, aligning a contemporary computational perspective with Spinoza’s insight that the complexity of experience reflects the complexity of the underlying system. Our claim is formulated at the level of capacity: a system with a larger and more structured repertoire of neural states can, in principle, support a richer and more differentiated range of possible experiences. This is consistent with the structural isomorphism we propose: the correspondence operates at the level of organizational principles, not at the level of individual elements.

### Internal susceptibility and reflexivity

Spinoza wrote “In proportion as the actions of a body depend more on itself alone, and as other bodies concur with it less in acting, so its mind is more capable of understanding distinctly.” (E2P13S) His insight can be understood as an early anticipation of access consciousness. Here, Spinoza emphasizes that a system’s increased autonomy, defined as self-determined activity less influenced by external factors, corresponds, under the attribute of Thought, to clearer and more distinct mental understanding. This notion resonates with IIT’s concept of intrinsic cause-effect power, where consciousness arises from a system’s capacity not only to produce effects but also to be modulated by its own internal states (Tononi et al. 2016).

Autonomy, derived from Greek roots meaning “self (autos)-law (nomos),” characterizes self-regulating systems capable of internally generated control. Such systems can monitor and modify their internal processes, supporting reflective awareness and access consciousness. This perspective aligns with the idiosyncrasy principle (Salti and Bergerbest 2022), which highlights that conscious experience depends on the unique physical structure of each individual, with internal organization underpinning self-awareness and introspective capacity. However, unlike emergentist frameworks like the idiosyncrasy principle, Spinoza maintains a monist ontology: consciousness is not a product of increasing complexity, but a manifestation of a single substance known through the attribute of thought.

What contemporary theory labels “access consciousness” corresponds, in the Spinozist framework, not to a separate kind of consciousness but to the reflexive dimension of any mental activity: the mind’s capacity to be affected by its own states. To connect this Spinozist framing with contemporary neuroscience, recurrent processing provides the relevant mechanism. Recurrent processing refers to feedback loops within and between brain regions, allowing information to be re-circulated and integrated rather than simply passed forward. Unlike feedforward processing, which transmits signals in a one-way manner from sensory input to higher-order areas, recurrent processing enables self-monitoring and recursive influence within neural networks (Lamme and Roelfsema 2000). This mechanism is central to theories of consciousness such as the Global Workspace Theory, which posits that conscious content emerges when information is widely broadcast and integrated through recurrent interactions (Baars 2005), and the IIT, which highlights the intrinsic cause-effect structure within a system as the basis for conscious experience (Tononi et al. 2016).

Adding to this synthesis, Recurrent Processing Theory (RPT) emphasizes the role of feedback interactions in distinguishing conscious from unconscious processing. RPT holds that unconscious functions are driven by the feedforward sweep of neural activity, while conscious functions depend on recurrent cortico-cortical interactions (Lamme 2018). In our monistic model, access consciousness is similarly linked to recurrent processing, which enables the brain’s self-influence and introspective capacity.

Within GNWT, this dynamic is captured by the notion of ignition—a nonlinear, threshold-based burst of recurrent neural activity that enables information to be broadcast across widespread cortical areas. Ignition marks the transition from a resting to an active brain state and underlies the moment when a stimulus enters awareness. Once this threshold is surpassed, recurrent processing sustains global activation even after the stimulus is removed, keeping the content available for reasoning, report, and introspection (Baars 2005, Moutard et al. 2015). Its nonlinear, threshold-based character points to broader dynamical principles in brain activity, a connection we return to when introducing critical brain dynamics below.

The importance of feedback and recurrent interactions extends to computational and neural models, where these mechanisms enable adaptive, dynamic responses rather than passive reactions. Feedback loops allow a system to adjust based on both internal and external information, enhancing control and flexibility. Reflecting this, some contemporary theorists argue that recurrent processing is not just a feature of complex cognition but a fundamental marker of consciousness itself (Lamme and Roelfsema 2000).

Within the attribute of extension (physical substance), access consciousness corresponds closely to these feedback mechanisms in neural systems. Recurrent processing enables the brain to exert influence over and monitor its internal states, facilitating introspection and self-regulation. This neurodynamical self-reference embodies Spinoza’s principle that greater bodily autonomy yields clearer mental understanding.

Consciousness does not simply increase with more recurrent connections; rather, it depends on the balance between feedforward and recurrent activity, which is optimized near criticality. At this point, recurrent processing is strong enough to support self-reflection and integration, while feedforward activity ensures responsiveness to external stimuli (Algom and Shriki 2025). This balance enhances the system’s capacity to modulate and monitor its own states, making integrated information maximally receptive and responsive to internal perturbations (Popiel et al. 2020). This dynamic is especially evident during altered states of consciousness, such as those induced by psychedelics, where individuals often report increased clarity and introspective depth (Toker et al. 2022). However, what underlies this dynamic is not merely the presence of recurrent connections, but the system’s autonomy and self-influence, which we conceptualize as internal susceptibility—the sensitivity of the system to its own prior states. In the manuscript describing the ConCrit framework (Algom and Shriki 2025), we mathematically demonstrate that internal susceptibility peaks at criticality, marking a transition where even small internal fluctuations can significantly shape ongoing activity. This reflects the essence of accessibility in consciousness: the brain’s recursive influence over itself.

Both dimensions of consciousness—experiential richness, rooted in entropy, and reflexivity, grounding in the system’s internal susceptibility, appear to reach their maximal expression near this critical point. We propose that, in complex organisms, criticality conditions correspond to consciousness as realized through the attribute of thought. At this point, internal susceptibility—the mind’s capacity to “understand distinctly”—and entropy—the ability to “perceive many things at once”—both reach their maximal expression, marking criticality as the regime where consciousness is most fully expressed.

### Fractals and brain organization: Enhancing adequate ideas

Spinoza stated: “By idea I understand a concept of the Mind that the Mind forms because it is a thinking thing. I say concept rather than perception, because the word perception seems to indicate that the Mind is acted on by the object. But concept seems to express an action of the Mind.” (E2D3&Ex). He further explained that an adequate idea is “an idea which, insofar as it is considered in itself, without relation to an object, has all the properties, or intrinsic denominations of a true idea.” (E2D4).

In Spinoza philosophy, an adequate idea—in the structural sense we specify below—is one whose form mirrors the common notions that are equally present in parts and whole (E2P38–39), an *adequate idea* is one that arises from the mind’s internal activity and accurately reflects the intrinsic nature of its object. These ideas are formed not through mere sensory impressions, which are considered as confused, but through a rational grasp of properties common to all things, such as extension, motion, and rest (E2P40S2). Crucially, Spinoza maintains that we necessarily form adequate ideas about properties that are common to all bodies and are equally present in both the parts and the whole (E2P38&39).

This concept finds a precise formal counterpart in scale-free (fractal) dynamics. Spinoza defines adequate ideas as those that grasp properties “common to all things” and “equally present in both the parts and the whole” (E2P38–39). Scale-free dynamics exhibit precisely this formal property: their organization is governed by generative rules that recur across scales, such that the same structural relations are preserved from local to global levels. This is not a superficial visual resemblance, but a genuine structural correspondence—both the formal structure of adequate ideas and scale-free dynamics are characterized by scale-invariant organization in which the universal is not abstracted away from particulars but realized through them.

Fractal dynamics are ubiquitous in nature, in trees, river networks, lightning, lungs, and vasculature, and are increasingly observed in neural systems. In the brain, fractal organization manifests as scale-invariant activity patterns across both space and time. Tools such as power spectral density analysis and IRASA (Irregular Resampling Auto-Spectral Analysis) reveal these self-similar dynamics by isolating the non-oscillatory, scale-free components of brain activity (Wen and Liu 2016, Gerster et al. 2022). These patterns may enable the brain to represent information in a way that is not limited to any specific scale, allowing for abstraction, integration, and generalization—hallmarks of adequate representation.

Importantly, these fractal structures often emerge within systems operating near criticality. According to Per Bak’s theory of *self-organized criticality* (Bak et al. 1987, Bak 2013), many natural systems spontaneously evolve toward critical states without the need for external fine-tuning. Bak argued that self-organized criticality is not just a feature of isolated systems but a fundamental principle of how nature operates, explaining why complex, scale-free patterns are found across all levels of organization, from sandpiles to ecosystems to neural networks. In this regime, systems exhibit rich, self-similar behavior across scales. If we adopt this view, then systems nearing criticality increasingly mirror the structure of nature itself. In Spinozist terms, under the attribute of extension, such systems become more isomorphic with the order of nature, and under the attribute of thought, this alignment is expressed as the structural adequacy of the corresponding ideas.

The universality of critical phenomena provides a further formal analogue: near criticality, system behavior becomes governed by universal laws largely independent of microscopic details—what physicists call universality classes. This emergence of shared structural regularities across diverse physical systems echoes Spinoza’s claim that adequate knowledge is grounded in properties common to all things and present in both part and whole (E2P38–39). A brain approaching this regime therefore increasingly instantiates the very structural regularities that, for Spinoza, are the content of adequate ideas.

Within the Spinozist framework, this structural correspondence provides a principled account of why scale-free dynamics correspond to adequate ideas. The account proceeds from Spinoza’s psycho-physical parallelism: every bodily state has a corresponding mental idea (E2P13). The question is not whether a mental idea exists—it always does—but what kind of idea it is: adequate or inadequate. Since scale-free organization is precisely the physical property that formally mirrors the content of adequate ideas, a brain exhibiting greater scale-free organization is one whose corresponding mental ideas are more likely to be structurally more adequate. The physical degree of scale-freeness maps, under the attribute of Thought, onto the degree of structural adequacy of the corresponding ideas. This is not a causal claim—scale-freeness does not produce structural adequacy through some intermediate mechanism. It is a parallelism claim: the same underlying reality, expressed under Extension as scale-free dynamics, is expressed under Thought as ideas that are structurally more adequate. No additional physical mechanism bridging neural dynamics and structural adequacy is needed or appropriate, because mind and body are not two things requiring a bridge, but one reality seen under two attributes. Proximity to criticality therefore enhances not only the brain’s computational efficiency but also, under the attribute of Thought, the structural adequacy of the ideas that correspond to its activity.

A caveat regarding the scope of this claim is in order. We use the term structural adequacy to designate the formal feature of an idea—cross-scale invariance, in correspondence with the common order of nature—that admits of dynamical characterization under the attribute of Extension as scale-free organization. Adequacy in Spinoza’s fuller sense (E3D2) involves this structural feature together with the causal origin of the idea within the mind: an idea that arises from the mind’s own activity—grasping common notions through reason—is adequate in the fullest Spinozist sense. Yet this formulation invites a puzzlement noted in the literature: as Spinoza states in E2P38–39, common notions are themselves formed when an external cause affects the human mind, so that ideas standardly treated as arising from the mind’s own activity turn out, at the level of their proximate cause, to be externally caused—raising the question of how such ideas could be adequate at all. (Bennett 1984, Della Rocca 1996, Marshall 2008) On the present account, this puzzlement dissolves: because structural adequacy tracks cross-scale invariance rather than causal history, an idea can be structurally adequate independently of whether its proximate cause is internal or external, so long as the resulting neural organization reflects the structure of the common order of nature—exactly the case of common notions. Adequacy in the fuller, causal-genetic sense (E3D2), in contrast, remains tied to causal origin: an idea whose proximate origin lies in external affection, as when the body is acted upon by external forces, may correspond under Extension to neural dynamics of considerable structural complexity, while remaining, under Thought, inadequate in this fuller sense. The parallelism we propose therefore holds between scale-free dynamics under Extension and structural adequacy under Thought; whether a given idea is also adequate in the fuller, causal-genetic sense depends on factors belonging to Spinoza’s broader theory of the affects (E3D2; Ethics III), which lie beyond the operational scope of the present framework.

This table outlines the core physical properties associated with brain dynamics near criticality and their corresponding mental dimensions, as interpreted through Spinoza’s parallelism. Each pairing illustrates how properties under the attribute of Extension relate to structural dimensions of conscious experience under the attribute of Thought, within a unified ontological framework, which providing a unified lens for understanding consciousness.

Although we have presented entropy, internal susceptibility, and scale-free dynamics as distinct properties of brain dynamics, this separation is artificial. These features co-emerge near criticality and are deeply interdependent: without recurrent connections, entropy is limited by a reduced range of internal configurations; without sufficient entropy and recurrence, scale-free dynamics cannot arise. At criticality, all three properties reach their maximal expression—not as independent variables that happen to peak together, but as constitutive aspects of a single dynamical regime. Under Spinoza’s parallelism, this convergence is not merely physical: the critical regime is itself a single psycho-physical event, conceivable under the attribute of Extension as an integrated pattern of neural dynamics and under the attribute of Thought as a unified conscious activity whose dimension—experiential richness, reflexivity, and structural alignment with nature’s common order—are co-emergent and inseparable. The conventional division between phenomenal and access consciousness thus dissolves not by theoretical fiat but as a consequence of the ontology itself: because the event is one, the dimensions through which it is grasped do not come apart. This convergence also places the present account within a specific interpretive tradition in Spinoza scholarship: because degree of consciousness here tracks degree of conceptual complexity—the integration of representation within the reflective system of the mind—the framework aligns closely with Della Rocca's (2003, 2008) and Nadler's (2008) readings, on which a mind’s power and the adequacy of its ideas scale together with the complexity of what it represents (cf. LeBuffe 2010, Sangiacomo 2011, Marrama 2017, Garrett 2018). The joint variation of these properties across conscious states is illustrated in Fig. 1.

**Figure 1:**
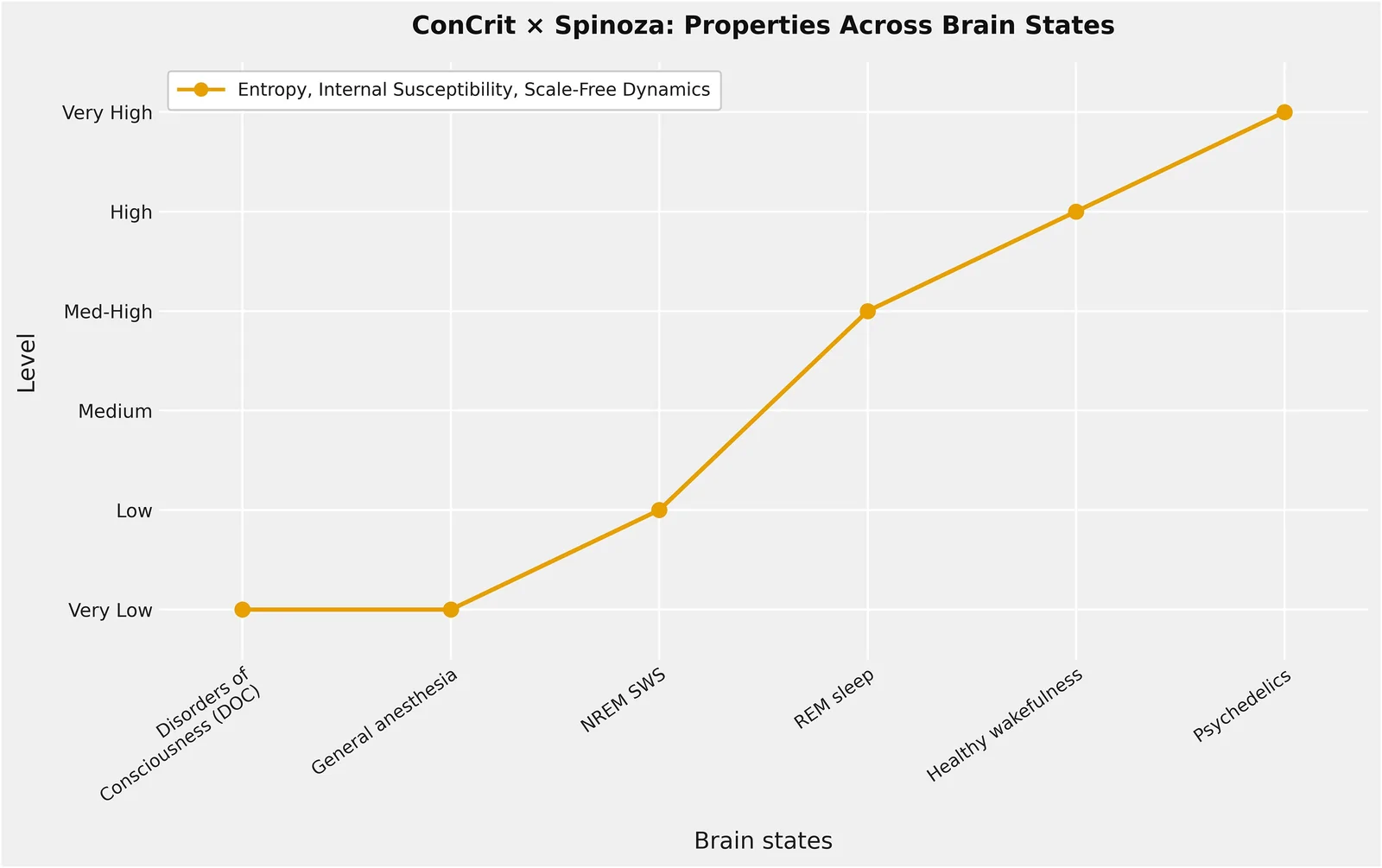
Properties of consciousness across brain states. This figure illustrates how entropy (experiential richness), internal susceptibility (reflexivity), and scale-free dynamics (structural alignment with the common order of nature) increase from DOC and anesthesia toward wakefulness and psychedelics, reflecting greater proximity to criticality. In the figure, psychedelics are shown at moderate doses, where they enhance these properties by bringing neural dynamics closer to the critical regime. At higher doses, however, psychedelics may push dynamics beyond the critical point into a supercritical regime (Bressloff et al. 2002, Bressloff 2005), which could reduce these properties as internally generated activity dominates perception (Algom and Shriki 2025).

### Critical brain dynamics and consciousness

An important concept in the proposed framework is that of criticality—the unique regime near a phase transition where systems exhibit distinctive computational properties. A growing body of empirical research supports the presence of critical dynamics in the brain, with evidence from neuronal avalanches, scale-invariant activity, and long-range correlations (Beggs and Plenz 2003, Shew and Plenz 2013, Shriki et al. 2013, O’Byrne and Jerbi 2022, Gervais et al. 2023, Hengen and Shew 2024). Recent findings further indicate that cortical activity remains near this critical regime specifically during conscious states (Toker et al. 2022, Gervais et al. 2023, Hengen and Shew 2024). Significant shifts away from this regime—either toward subcritical or supercritical dynamics—are associated with diminished consciousness, as observed in states such as deep sleep, general anesthesia, and disorders of consciousness (DOC) (Fekete et al. 2021, Toker et al. 2022, Gervais et al. 2023, Maschke et al. 2024). In contrast, movement toward the critical point—such as that induced by psychedelic substances—has been linked to heightened richness and complexity of conscious experience, though only few studies have examined this (Gervais et al. 2023).

This regime is characterized by high susceptibility, long-range correlations, and scale invariance, offering significant computational advantages by balancing flexibility with stability and enabling efficient processing, transmission, and integration of information. While entropy has often been highlighted as a correlate of conscious richness, it represents just one feature of criticality. Other hallmarks of criticality confer distinct computational advantages. These include expanded dynamic range, enabling neural circuits to respond to both weak and strong inputs (Kinouchi and Copelli 2006, Shew et al. 2009, Shew and Plenz 2013), enhanced sensitivity to stimuli (Kinouchi and Copelli 2006), and increased information transmission and storage (Shew and Plenz 2013, Fagerholm et al. 2015). Additionally, scale-invariant dynamics facilitate long-range integration across spatial and temporal scales, linking local computations to global functions (He 2014, Hesse and Gross 2014, Fekete et al. 2018). Together, these properties enable the brain to integrate information across multiple scales while remaining responsive to change—capacities essential for sustaining coherent, adaptive, and rich conscious states.

Importantly, this emphasis on multi-scale organization is broadly consistent with frameworks that highlight the temporo-spatial structure of brain activity. For instance, the TTC proposes that consciousness is shaped by the interaction between spontaneous and stimulus-related activity across multiple temporal and spatial scales, including mechanisms such as nestedness, alignment, and expansion (Northoff and Zilio 2022). These mechanisms describe how neural activity is organized across time and space in a way that systematically relates to different dimensions of consciousness. Within this context, criticality may provide a dynamical regime that supports or constrains such temporo-spatial organization, offering a potential basis for the emergence of the multi-scale structure emphasized in TTC.

This also resonates with the GNWT, particularly with the mechanism of ignition described above. Such threshold dynamics map naturally onto criticality: when ongoing activity is poised near a critical point, small perturbations can trigger large-scale coordinated activation, making ignition events more likely (Moutard et al. 2015). In this way, GNWT’s ignition provides a mechanistic expression of how critical dynamics support the accessibility and integration of conscious content.

Although the precise control parameters governing neural criticality remain under investigation, candidates include neuronal excitability, synaptic gain, network connectivity, and neuromodulatory tone (Algom and Shriki 2025). Unlike classical systems governed by a single transition, the brain may exhibit multiple forms of criticality, such as neuronal avalanches, edge-of-chaos dynamics, and pattern formation, each associated with distinct computational roles. These regimes may co-occur or shift dynamically based on neuromodulatory input or sensory context. Moreover, due to its structural heterogeneity, the brain may operate within a Griffiths phase: a regime of extended near-critical behavior that preserves the computational benefits of criticality without requiring precise tuning (Moretti and Muñoz 2013). This perspective provides a powerful lens for understanding how the brain balances stability and flexibility in support of consciousness.

Near criticality, the key properties we have interpreted through Spinoza’s framework—entropy, complexity, internal susceptibility, and scale-free dynamics—reach their maximal expression. This positioning makes critical brain dynamics theory a compelling neurodynamical framework for describing the physical organization of brain activity associated with conscious experience, proposing that neural networks operate near critical points akin to phase transitions (O’Byrne and Jerbi 2022, Hengen and Shew 2024). This concept originates in statistical physics, where criticality describes the unique state a system occupies at the boundary between distinct phases, such as when a ferromagnet loses its magnetization at the Curie point. While often mentioned alongside various measures of brain complexity, criticality is not merely another metric. Rather, it is a distinctive form of collective behavior that emerges in systems composed of many interacting parts. It may be the primary mechanism by which nature generates complexity (Langton 1990, Bak 2013). Near a critical point, neural activity becomes both highly variable and deeply structured. This regime characterizes how neural dynamics become both highly variable and structured, and how they systematically co-vary with conscious states (Algom and Shriki 2025).

To bring these insights together, we recently proposed the ConCrit framework: Critical Brain Dynamics as a Unifying Mechanistic Framework for Theories of Consciousness (Algom and Shriki 2025). Within candidate biological systems in which consciousness can be independently assessed, proximity to criticality is predicted to covary with the level or presence of consciousness. The weak ConCrit hypothesis holds that proximity to criticality increases the likelihood or intensity of conscious experience. The strong version advances a more stringent claim: that criticality is both necessary and sufficient for consciousness—such that any significant change in conscious state must correspond to a measurable shift in the system’s proximity to critical dynamics. At the same time, we do not claim that criticality exhaustively defines all systems capable of consciousness, nor that it constitutes the only possible route to conscious experience. Rather, it identifies a class of dynamical regimes that are strongly associated with, and may facilitate, variations in conscious states.

Importantly, this framework is not intended to define the full class of systems capable of subjective experience. Rather, it operates within an empirical domain consisting of biological systems, such as the human brain, in which the presence or absence of consciousness can be independently assessed through behavioral and clinical criteria. In this sense, the phrase “systems capable of subjective experience” serves as a scope condition rather than a formally specified category.

While ConCrit provides a neurodynamical account of neural dynamics, Spinoza’s philosophy offers a complementary ontological perspective. Under the attribute of Extension, near-critical dynamics describe neural processes, while under the attribute of Thought, these same processes are expressed as conscious experience. From this perspective, the relationship between neural dynamics and experience is not mechanistic in the standard causal sense. Rather, they are two aspects of a single underlying process, and no additional physical mechanism is required to transform neural activity into consciousness. In this way, ConCrit situates empirical findings within a coherent metaphysical framework.

Within this domain, the framework generates testable predictions: across transitions between brain states, including wakefulness, sleep, anesthesia, DOC, and psychedelic states, proximity to criticality is expected to systematically covary with independently assessed levels of consciousness.

### Spinoza and the brain: consciousness properties and predictions in the ConCrit framework

#### Entropy, internal susceptibility, and self-similarity across brain states *a Spinozist view of consciousness-related properties near criticality*

The ConCrit framework advances testable predictions that can stand independently of any metaphysical stance, but their broader significance becomes clearer when viewed through Spinoza’s philosophy. ConCrit, by design, does not single out specific features of criticality as most relevant for consciousness, focusing instead on proximity to criticality as the unifying mechanism. By adopting Spinoza’s perspective, we move beyond mechanism alone: near-critical dynamics are not only computationally advantageous but constitute the parallel expressions of one unified substance. Under the attribute of Extension, the increases in entropy, internal susceptibility, and scale-free organization that occur as the system approaches criticality manifest as enhanced physical organization; under the attribute of Thought, they manifest as ideas with greater experiential richness, reflexivity, and structural adequacy.

What is gained from this synthesis is conceptual: in brief, the ConCrit framework proposes that critical brain dynamics may correspond to consciousness, such that in systems capable of subjective experience and capable of exhibiting a critical phase transition, approaching the critical point is expected to correspond to heightened consciousness, whereas significant deviations toward subcritical or supercritical dynamics are associated with unconscious or altered states (Algom and Shriki 2025); Spinoza’s framework then explains why these very dynamics correspond to subjective experience. Spinoza dissolves the apparent gap between neural mechanism and the experience, showing them as two aspects of the same reality. Thus, the approach to criticality is simultaneously the brain’s physical trajectory toward optimal organization and the mind’s progression toward richer experience. This integration transforms ConCrit from a purely scientific hypothesis into a philosophically grounded framework in which empirical predictions and metaphysical insight mutually reinforce one another.

ConCrit predicts that brain regions closer to criticality contribute more strongly to consciousness (Algom and Shriki 2025). Higher-order cortical areas, which integrate information across broad spatial and temporal scales, are plausible candidates. The current paper adds that, from Spinoza’s perspective, these regions instantiate three properties maximized near criticality—entropy, internal susceptibility, and scale-free dynamics; under the attribute of Thought, their activity is expressed as fully formed conscious ideas.

By contrast, lower-order bodily activity operates further from criticality and expresses these properties more weakly. Under the attribute of Thought, the corresponding ideas remain inadequate and poorly integrated into the reflexive organization of the whole mind—not because they lack mental expression altogether (every bodily state, on Spinoza’s parallelism, has its corresponding idea), but because they fail to reach the structural organization that, under the attribute of Thought, is required for reflexive awareness. This perspective addresses what Spinoza scholars have termed the ‘pancreas problem’: lower-order bodily activities do have mental correlates, but these correlates do not attain the integrated, self-referential organization that characterizes reflexively accessible conscious experience. Higher-order areas closer to criticality, by comparison, encode content that becomes conscious because it corresponds, under Thought, to ideas formed with maximal clarity (Bennett 1984, Della Rocca 1996: 9, Nadler 2008, Marrama 2017, Toth 2026).

Critical dynamics also accord with the mechanisms proposed by Higher-order Theories (HOTs), which argue that a mental state becomes conscious only when re-represented by a higher-order cognitive state- a thought about a thought (Fleming 2020, Seth and Bayne 2022). Near criticality, higher-order areas are especially sensitive to the activity of other brain regions, enabling them to amplify and re-represent signals from lower levels, as required by HOTs.

This view also helps explain why different manipulations of brain dynamics have such distinct experiential consequences. For instance, viewed through the lens of Spinoza, the current paper offers insight into why psychedelics may be considered “mind-revealing.” By expanding the proximity to criticality across large-scale brain networks, psychedelics may increase the expression of entropy, internal susceptibility, and scale invariance in broader regions. Under the attribute of Thought, this expansion allows more content to become accessible to consciousness, consistent with the intensified, expansive, and often transformative nature of the psychedelic experience. Conversely, anesthetics induce a loss of consciousness by shifting brain networks away from criticality—either into subcritical or supercritical regimes—suppressing the physical expression of entropy, internal susceptibility, and scale invariance. Under the attribute of thought, this diminishes the mind’s capacity to perceive and integrate information, making fewer ideas accessible and ultimately disrupting the unified experience of consciousness.

The levels of entropy, internal susceptibility, and self-similarity vary with the brain’s position- subcritical, critical, or supercritical—along multiple axes of criticality, each reflecting a distinct type of phase transition (Algom and Shriki 2025). This view offers clear empirical targets for future research: by measuring these properties across brain states, we can test whether near-critical dynamics are indeed the physical dimension (under the attribute of Extension) of consciousness (under the attribute of Thought). Such investigations would not only validate the ConCrit framework but also illuminate the deep connection between neural dynamics and the structure of consciousness itself.

## Discussion

This paper presents Spinoza’s theory of consciousness, integrated with the empirical perspective of critical brain dynamics, along with mathematical and physical principles essential to the main computational theories of consciousness. We propose it as a paradigmatic alternative to prevailing approaches in consciousness research. Rather than treating consciousness as either an emergent product of physical mechanisms or as fundamentally distinct from them, we adopt a monistic perspective grounded in Spinoza’s ontology. In this view, everything that exists does so in two parallel aspects—conscious and physical—as one unified substance. Consciousness, then, is the expression of this reality under the attribute of *Thought*, whereas the same underlying reality, under the attribute of *Extension*, is expressed as a neurophysical mechanism.

Building on this monistic perspective, we can now describe how consciousness manifests both as physical dynamics and as subjective experience. Instead of treating consciousness and its physical underpinnings as separate explanatory domains, our framework demonstrates how they can be described in parallel. As we have shown, consciousness can be described in terms of the physical organization of brain activity near criticality—characterized by heightened entropy, internal susceptibility, and scale-free dynamics—features that characterize brain activity when it operates near criticality. According to our suggested framework, this is the description of consciousness in terms of the order and connection of things. At the same time, these physical, chemical, and biological processes—which follow logical and mathematical principles—are experienced as thoughts, awareness, perception, intellect, and reasoning. This is their expression within the order and connection of ideas. As Spinoza stated in Ethics (E2P7) as: “The order and connection of ideas is the same as the order and connection of things.” Natural principles such as those expressed in mathematics and physics are, in Spinoza’s view, not abstract entities that exist in some Platonic realm, nor emergent properties of physio-chemical processes. Rather, they are the very structure of reality—experienced as phenomena in the universe and the human body—and analyzed through science. The added value of the present framework is therefore not to propose a direct physical mechanism of experience, but to identify a well-defined class of neural dynamics and to show how these dynamics can be coherently interpreted within a Spinozist ontology that dissolves the explanatory gap.

The same logic applies to the case of neuronal correlates of consciousness (NCC). The physical mechanism—whether located in the brain, the body, or the broader physical environment—is not a substance distinct from experience. Nor is experience a mysterious, abstract, disembodied entity separate from physical being. From this perspective, the epistemological relationship between neural and experiential aspects of consciousness (which are ontologically one) should not be described as a *correlation* but rather as *aspects*. According to the *Oxford English Dictionary* (n.d.), “correlation” refers to the “mutual relation of two or more things (implying intimate or necessary connection).” Within the Spinozist framework, however, there are not two distinct things, but one reality that appears and is described through two parallel orders and terminologies: those of *Thought* and of *Extension*.

Beyond the specific case of NCC, this framework also addresses a deeper challenge in consciousness research. It seeks to resolve a central conceptual impasse: the enduring explanatory gap and the lack of a coherent metaphysical foundation. By revisiting the historical and logical origins of this gap, we argue that it stems from dualistic assumptions that artificially separate the mental from the physical. The ConCrit-Spinoza perspective reframes this division, suggesting that subjective and objective phenomena are not causally linked across metaphysical domains, but are instead parallel expressions of one unified reality. This ontological interpretation clarifies why certain brain states, such as those induced by psychedelics, correspond to expanded conscious experience as their proximity to criticality is enhanced, while others, like anesthesia, diminish it by moving the system away from criticality. In this view, consciousness is not a mysterious byproduct of complexity but a coherent and intrinsic manifestation of nature’s organization, intelligible and accessible to both empirical and philosophical investigation. This perspective also resolves another central challenge: the divisions often drawn within consciousness itself. Distinctions such as phenomenal *versus* access consciousness remain methodologically useful, but in the ConCrit-Spinoza framework they are not treated as ontologically separate kinds of consciousness; rather, they are understood as complementary dimensions of the same underlying process. Entropy, internal susceptibility, and scale-free dynamics co-emerge at criticality, and it is from this shared dynamical foundation that both the richness of subjective experience and the accessibility of mental content arise. What has long been treated as two kinds of consciousness thus appears, in the ConCrit-Spinoza framework, as two facets of a unified reality grounded in critical brain dynamics.

While the ConCrit framework yields empirically testable predictions on its own, integrating it with Spinoza’s philosophy offers a deeper explanatory perspective. From this combined view, proximity to criticality is not merely a computational advantage that supports consciousness, but the physical expression—under the attribute of Extension—of the same phenomenon that, under the attribute of Thought, is conscious experience. This perspective explains why higher-order cortical regions, which integrate information across broad scales and operate closer to criticality, support more unified and accessible experiences: their proximity to criticality makes the content they encode accessible to consciousness, as it corresponds—under the attribute of Thought—to higher degrees of reflexive integration. In this sense, ConCrit provides a mechanistic account of the neural dynamics associated with consciousness, while Spinoza’s framework explains how these dynamics and subjective experience are two aspects of a single underlying process.

In addition to organizing empirical results, the framework contributes theoretically by clarifying the relationship between leading theories of consciousness. IIT, the EBH, and the GNWT each highlight essential features of consciousness, but they generally lack a unifying ontological framework. IIT emphasizes information integration, GNWT focuses on global broadcasting, and EBH links entropy to experiential richness. Despite their value, these theories often rest on implicit metaphysical assumptions and leave unaddressed the deeper question of *why* such mechanisms should be accompanied by subjective experience, thereby diminishing their explanatory power. We propose a new perspective in which the problem—relevant solely to the dualistic assumption—does not arise at all. Like other leading theories of consciousness, this framework operates within a set of candidate systems and focuses on identifying principles governing variations in conscious states, without requiring a prior formal solution to the problem of consciousness attribution.

The ConCrit-Spinoza framework revisits these theories while simultaneously addressing their philosophical gaps. It preserves their structural insights but reframes their significance within a non-dualistic model. IIT’s concepts of cause-effect power and intrinsic existence align with entropy and internal susceptibility under Extension; GNWT’s ignition dynamics—threshold-based, nonlinear bursts of recurrent activation—naturally arise near criticality, where internal susceptibility is maximal and small perturbations can trigger large-scale state transitions; and EBH’s link between entropy and conscious richness echoes Spinoza’s claim that greater bodily complexity supports a richer mind. These mappings, introduced earlier, are here reframed not as mere functional correlations but as manifestations of the same structure viewed under different attributes—Extension and Thought.

The present framework is closely related to, and partially convergent with, the TTC, which similarly aims to provide an integrative account linking neural activity and subjective experience. Both approaches emphasize structural principles that span neural and experiential domains and move beyond strictly reductionist interpretations of neural correlates. TTC identifies temporo-spatial dynamics as a “common currency” mediating this relationship, focusing on how the brain’s intrinsic organization shapes conscious experience (Northoff and Zilio 2022).

The current framework can be understood as complementary to TTC in two key respects. First, while TTC characterizes the form of neural organization in temporo-spatial terms (Northoff and Zilio 2022, Northoff et al. 2025), ConCrit proposes a candidate dynamical regime—proximity to criticality—that may generate and regulate such organization. Second, while TTC primarily formulates the neural-phenomenal relationship in neuroscientific and empirical terms (Northoff and Zilio 2022, Northoff et al. 2025), the present work introduces a Spinozist ontological perspective, according to which neural and experiential phenomena are identical aspects of a single underlying process. Together, these perspectives suggest that convergence across independently developed frameworks may point to shared underlying principles of consciousness.

Extending these theoretical mappings, one can speculate that the brain’s complex dynamics might reflect not only its internal organization but also broader structural principles of the universe. An intriguing extension considers the possibility that the brain’s fractal dynamics resonate with the fractal structure of space–time itself. In his theory of scale relativity, Nottale proposed that if we abandon the assumption of differentiability, space–time must be understood as fundamentally fractal, with quantum behavior emerging as a manifestation of this geometry rather than from separate axioms (Nottale 1993). On this view, if space–time itself is fractal, then as brain dynamics become more fractal, they become increasingly isomorphic with that underlying structure. In this context, the concept of complexergy, a proposed measure of hierarchical self-organization, may offer a way to quantify the degree to which neural dynamics align with such universal structural principles. Higher levels of complexergy could thus correspond to more integrated and enriched states of consciousness. Though speculative, this perspective aligns with our broader proposal: that consciousness emerges not merely from local complexity, but from the brain’s increasing isomorphism with the universal principles governing nature across scales. This recalls the microcosm–macrocosm principle common among pre-modern thinkers and evident in Spinoza’s own writings (Spinoza and de Spinoza and Curley 1985: 462), and resonates with contemporary science, which assumes that humans are not exempt from the physical and logical laws that govern the rest of the universe.

Drawing on the speculative connections between brain dynamics and universal structural patterns, the framework also opens new philosophical and empirical avenues for future research. Theoretically, the relationship between scale-free brain dynamics and Spinoza’s notion of *adequate ideas* warrants further elaboration. Empirically, there is a need for new tools to identify and quantify different forms of criticality across brain states and to evaluate their specific roles in generating conscious experience. Furthermore, it remains an open question whether artificial systems exhibiting critical dynamics can instantiate even minimal forms of consciousness under this framework. Since consciousness, in our view, depends not only on dynamic regimes but also on the underlying substrate, future work must clarify the biological and structural conditions necessary for experience. This inquiry lies beyond the scope of the current paper.

The ConCrit-Spinoza framework represents more than a reinterpretation of existing theories; it proposes a fundamental paradigm shift in consciousness research. By recognizing that mind and body are not separate entities requiring a bridge but rather two aspects of one reality, we can move beyond the conceptual impasses that have long plagued the field. This perspective aligns consciousness research with a robust philosophical foundation while maintaining rigorous empirical standards, potentially guiding both theoretical development and experimental design in the years to come.

This shift may realign the study of consciousness with a coherent philosophical foundation, providing guidance for future theories and experiments alike.

## Conclusion

This paper has presented the ConCrit-Spinoza framework, a unified account of consciousness that integrates Spinoza’s monistic ontology with the empirical theory of critical brain dynamics. In this view, mind and body are not separate entities requiring a bridge but parallel expressions of a single reality, and the hard problem does not arise when subjective experience and physical processes are reframed as attributes of the same substance. The framework identifies measurable properties of brain dynamics—entropy, internal susceptibility, and scale-free organization—that correspond to distinct aspects of conscious experience and reach their maximal expression near criticality. These features also reconcile major theories of consciousness, showing how IIT’s cause-effect power, GNWT’s global broadcasting, and EBH’s entropic richness describe complementary facets of the same critical regime. From this integration follow concrete predictions: systematic variation of these measures across brain states, higher-order cortical regions operating closer to criticality than lower-order areas, and correlations between criticality and conscious accessibility. These predictions provide empirical targets for testing, from DOC to psychedelic states, and raise open questions about the conditions under which artificial systems could instantiate minimal forms of consciousness. By aligning philosophical insight with neuroscience, this framework offers a paradigm shift in consciousness research, situating consciousness not as an inexplicable mystery or illusion but as a natural phenomenon that is the parallel manifestation—under the attribute of Thought—of the organizational principles of criticality—intelligible to both empirical and philosophical investigation and understanding.

## Data Availability

No empirical data were generated or analyzed for this study.
